# Muscle function around the hip after operation for Fibrous Dysplasia of Proximal Femur with Varus Deformity

**DOI:** 10.12669/pjms.39.5.6987

**Published:** 2023

**Authors:** Zhigang Lang, Xu Zhang

**Affiliations:** 1 Zhigang Lang, Department of Orthopedics, Sichuan Orthopedic Hospital, Chengdu 610041, Sichuan, China; 2 Xu Zhang, Department of Orthopedics, Sichuan Orthopedic Hospital, Chengdu 610041, Sichuan, China

**Keywords:** Isokinetic muscle strength, Proximal femur, Fibrous dysplasia, Varus deformity, Orthopaedic osteotomy

## Abstract

**Objectives:**

This study aimed to analyse the clinical effect of surgery for patients with fibrous dysplasia (FD) of the proximal femur with varus deformity, detect the muscle function around the hip joint.

**Methods:**

This is a retrospective study. A total of 20 patients who underwent operation for FD of the proximal femur with varus deformity at West China Hospital, from January 2018 to October 2022 were included in this study. The peak torque (PT) of muscle strength during abduction, adduction, flexion and extension of bilateral hip joints was measured by an isokinetic muscle test and training system associated with the comparison of bilateral hip joints, to evaluate the function of muscles around the hip joint after operation.

**Results:**

The difference between the length of the affected limb and the healthy side was 1~2 cm in seven cases and <1 cm in 13 cases. The force line showed no abnormality in 15 affected limbs, which deviated from the center of the knee joint by 3-5 mm in five cases. The average PT during abduction of hip joints on the affected side of 20 patients was significantly lower than that on the healthy side, which was also reduced evidently during adduction and flexion of hip joints on the affected side compared with that on the healthy side.

**Conclusions:**

After effective recovery of the force line and length of the affected limb in patients with FD of the proximal femur with deformity, functional abnormalities of muscles are still present to a certain extent.

## INTRODUCTION

Patients with limb deformity may have different degrees of muscular imbalance and soft tissue dysfunction, in addition to bone deformity. Orthopaedic surgery can correct limb alignment and restore length, but it cannot timely improve the function of muscle and soft tissue. Abnormal muscle function can directly affect the muscular balance of limbs and the stability of gait. Clinically, muscle strength (muscle strength) is a common option for evaluating muscle function. Traditionally, muscle strength is measured by manual muscle testing (MMT), which artificially divides the muscle strength into grades 0-5 (Grade-0 being the worst and grade five being normal). In addition, the isokinetic muscle strength training and testing system is a new detection and evaluation method, which can provide data-based muscle function indicators. It is more accurate and intuitive with regard to the traditional muscle strength measurement, which can meet the requirements of evaluating limb function.

At present, the isokinetic muscle strength training and testing system has been extensively applied in the diagnosis and treatment of joint diseases, injuries and postoperative rehabilitation, with the achievement of some research results.^1^,^2^ Nevertheless, no report on the isokinetic study of fibrous dysplasia (FD) of the proximal femur with varus deformity has been found. Thus, this study included 20 eligible patients with FD of the proximal femur with deformity after surgery for isokinetic muscle strength testing of bilateral hip joints. Furthermore, our study compared the muscle strength around the hip joint, analyzed the characteristics of muscle strength and evaluated the postoperative muscle function around the hip to promote gait restoration.

## METHODS

This is a retrospective study. This study included 20 patients who were admitted to the Department of Orthopedics, West China Hospital, Sichuan University, to be operated for unilateral FD of the proximal femur with varus deformity by using orthopaedic osteotomy from January 2018 to October 2022

### Ethical Approval:

The study was approved by the Institutional Ethics Committee of West China Hospital, Sichuan University (No.: 2017-6-30-1; Date: June 30, 2017), written informed consent was obtained from all participants, permission was taken to publish the photographs in the write up.

### Inclusion criteria:

- Patients aged between 10 and 35 years.
- Patients with coxa varus or femoral varus deformity caused by unilateral FD of the proximal femur (without contralateral abnormality).
- Patients with unilateral lower limb force line abnormality indicated by imaging and lesion located primarily in the proximal femur.
- Patients with orthopaedic surgery for >one year.

### Exclusion criteria:

- Patients with congenital and non-neoplastic femoral lesions complicated with deformity.
- Patients with congenital pelvic and spinal deformities.
- Patients with serious complications, such as fracture of internal fixation and infection of osteotomy end.
- Patients who cannot cooperate with the study for any reason.

Finally, 20 eligible cases were included for further study and Table-I shows the baseline data of the enrolled patients. The basic parameters of the human body (height, weight, knee width, ankle width, foot width, etc.) were measured, associated with the measurement of the force line and length of the affected limb by imaging examination. Based on the classification standard of proximal femoral FD in West China Hospital of Sichuan University^3^, the enrolled patients were divided into Group-A (femoral varus group), Group-B (mild coxa varus group) and Group-C (severe coxa varus group).

**Table-I:** Baseline data of the enrolled patients (n = 20).

Indexes	Distribution Range
Cases	20
Gender (male/female)	11/9
Age (years)	10-35 (21.6 ± 3.4)
BMI (kg/m^2^)	17.2-29.5 (24.3 ± 1.7)
Operative side (left/right)	10/10
Time after operation (months)	13-65 (35.6 ± 5.2)

In Group-A, patients had no abnormality in the neck-shaft angle of the affected limb and the varus angle of the proximal femur was >20°, with or without the decrease of the cortical bone strength of the proximal femur (cortical bone thickness of <50%). In Group-B, the neck-shaft angle of the affected limb was between 90° and 120°, with or without the decrease of the cortical bone strength of the proximal femur. On the contrary, in Group-C, the neck-shaft angle was less than 90°, with or without the decrease in the cortical strength of the proximal femur (Table-II). No significant difference in baseline data was found among the three groups (*p* > 0.05, Table-III).

**Table-II:** General data of the enrolled patients.

Cases	Age (years)/gender	Follow-up (month)	Diagnosis	Angle of varus (°)
*Group-A*				
1	24/male	50	FD of the proximal right femur with femoral varus	29.2
2	28/male	42	FD of the proximal left femur with femoral varus	28.4
3	20/female	13	FD of the proximal left femur with femoral varus	25.1
4	20/male	20	FD of the proximal left femur with femoral varus	29.5
5	32/female	51	FD of the proximal right femur with femoral varus	26.8
6	24/male	36	FD of the proximal right femur with femoral varus	30.2
*Group-B*				
1	16/female	29	FD of the proximal left femur with coxa varus	17.5
2	34/female	54	FD of the proximal right femur with coxa varus	19.7
3	24/male	52	FD of the proximal right femur with coxa varus	27.3
4	13/male	24	FD of the proximal right femur with coxa varus	22.9
5	38/male	42	FD of the proximal right femur with coxa varus	19.6
6	21/female	22	FD of the proximal right femur with coxa varus	18.5
7	22/female	14	FD of the proximal left femur with coxa varus	20.4
8	14/male	28	FD of the proximal left femur with coxa varus	25.6
*Group-C*				
1	22/female	32	FD of the proximal left femur with coxa varus	48.2
2	31/female	37	FD of the proximal right femur with coxa varus	46.3
3	12/male	40	FD of the proximal left femur with coxa varus	45.3
4	17/male	13	FD of the proximal left femur with coxa varus	46.8
5	27/male	60	FD of the proximal left femur with coxa varus	42.4
6	15/female	17	FD of the proximal right femur with coxa varus	41.2

**Table-III:** Comparison of baseline data of three groups of patients.

Indexes	Group-A	Group-B	Group-C	P
Cases	6	8	6	0.658
Gender (male/female)	4/2	4/4	3/3	0.274
Age (years)	25.03±6.49	22.65±8.62	20.75±7.12	0.124
BMI (kg/m^2^)	22.13±1.12	20.13±0.98	23.13±1.54	0.574

MMT was performed to measure the muscle strength around the bilateral hip joints of the included 20 patients. Isokinetic muscle strength testing system (ISOMED2000, Germany) was then used to test the isokinetic muscle strength and peak torque (PT) of bilateral hip joints. Before the test, patients were informed to carry out adaptability training on the healthy side. After mastering the essentials of force, the measurement was started from the healthy side and then to the affected side.

In measuring the muscle strength of the hip joint during flexion and extension, patients were informed to keep their supine position on the testing device, and the waist shall be fixed on the bed surface. During the test, a hip adapter was used to fix the front side of the lower thigh. The adaptability exercise was carried out firstly after laser positioning. Testing was conducted at the angular velocity of 60°/s using the active mode, ranging from 0° to 90°. Four groups were measured on each side, and flexion and extension were repeated 10 times in each group. The PT values of isokinetic muscle strength testing during flexion and extension in each group were recorded. The maximum value of the four groups of parameters was obtained and compared with that of the healthy side.

**Fig.1:**
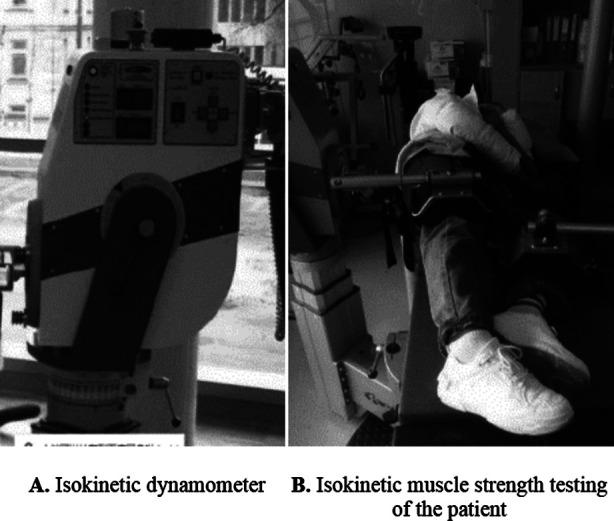
Isokinetic muscle strength testing device and operation diagram.

Subsequent measurement was performed to determine the PT of the muscle strength of bilateral hip joints during abduction and adduction. These patients were adjusted to keep their lateral position with the limb to be measured on top, and the hip adapter was fixed on the outside of the lower end of the thigh to be measured. The testing was conducted at the angular velocity of 60°/s using the active mode, ranging from 30° to 45°. Four groups were measured on each side, and the movement of abduction and adduction was repeated 10 times in each group (Fig.1). Afterwards, the PT values of isokinetic muscle strength testing during abduction and adduction in each group were recorded, followed by the calculation of the maximum values to compare with those of the healthy side.

Isokinetic muscle strength testing parameters included PT, PT/BW, total power and average power. Amongst these parameters, the total power and average power are generally used to measure the endurance of muscle to fatigue, particularly in athlete testing. PT and PT/BW are common parameters for testing muscle strength, which is suitable for the analysis of muscle strength in the present study.

**Fig.2:**
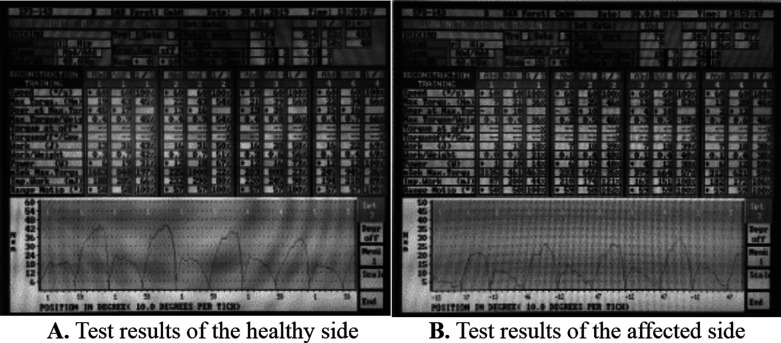
Real time torque curve diagram of the subjects (red, adduction; blue, abduction).

PT refers to the maximum torque value generated by the joint under the premise of fixed acceleration and range of motion, which is the peak value on the torque curve (N*m, Fig.2). PT is considered to be the gold index of isokinetic muscle strength testing, which represents the maximum muscle strength during muscle contraction.^4^

PT/BW is the PT value per unit weight (N*m/kg), which is used for muscle strength comparison amongst different subjects.^5^

### Statistical analysis:

SPSS20.0 was used for statistical processing. Measurement data were expressed as mean ± standard deviation (`c ± *s*) and compared by t-test amongst groups, and the classified counting data were analyzed by analysis of variance. *P* < 0.05 indicated statistically significant difference.

## RESULTS

Results of follow-up: All 20 patients were followed up for 13-65 months, with an average of 35.6 months. Change in the length of the affected limb: The difference between the length of the affected limb and the healthy side was 1-2 cm in seven cases and <1 cm in 13 cases. Force line results: The force line showed no abnormality in 15 affected limbs, which deviated from the center of the knee joint by 3-5 mm in five cases and the neck-shaft angle recovered from the preoperative average of 92.5° ± 3.14° to the postoperative average of 128.6° ± 2.56°. Measurement results of MMT: Based on the results of MMT in 20 patients, three patients had a muscle strength of Grade-4 during hip abduction, and another three patients had a muscle strength of grade four during abduction and extension. In addition, the muscle strength around the hip joint of other patients reached Grade-5.

**Fig.3:**
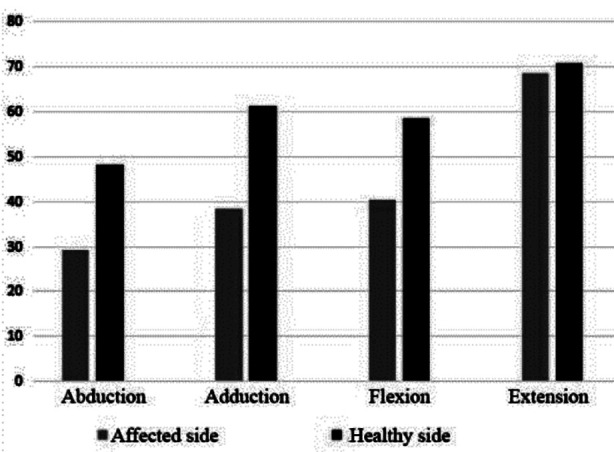
Comparison of PT between the affected side and healthy side (N*m).

In 20 patients, the average value of PT of the hip joint during abduction was 29.2 ± 3.7 N*m on the affected side, which was significantly lower than that on the healthy side of 48.1 ± 2.3 N*m (*p* = 0.013) and evidently reduced during adduction (39.3 ± 5.8 N*m vs 61.2 ± 5.4 N*m, *p* = 0.026) and flexion (40.3 ± 2.8 N*m vs 58.2 ± 3.2 N*m, *p* = 0.032) of hip joints. In addition, no significant difference in PT during extension of hip joints was observed between the affected side and healthy side (68.5 ± 3.5 N*m vs 70.8 ± 4.2 N*m, *p* = 0.231; Table-IV and Fig.3).

**Table-IV:** Comparison of the PT of hip joints between the affected side and healthy side (n = 20).

Isokinetic motion mode	Abduction (N*m/kg)	Adduction (N*m/kg)	Flexion (N*m/kg)	Extension (N*m/kg)
Affected side	29.2 ± 3.7	39.3 ± 5.8	40.3 ± 2.8	68.5 ± 3.5
Healthy side	48.1 ± 2.3	61.2 ± 5.4	58.2 ± 3.2	70.8 ± 4.2
*p* Value	0.013	0.026	0.032	0.174

**Fig.4:**
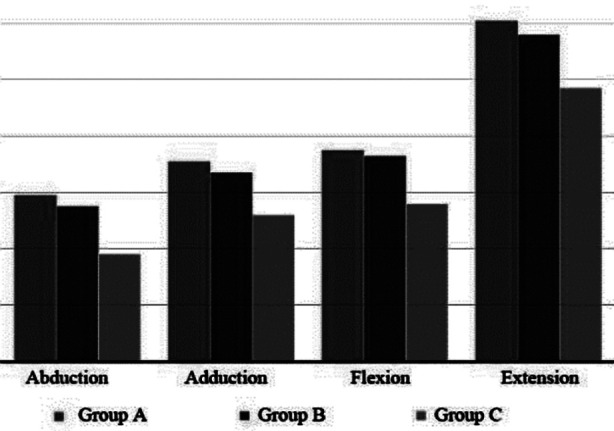
Comparison of PT/BW amongst the three groups (N*m/kg).

As shown in Table-V and Fig.4, no significant difference in the average value of PT/BW during abduction, adduction, flexion and extension of hip joints was observed between Group-A and Group-B (*p*> 0.05).

**Table-V:** Comparison of the PT/BW of the hip joint in three groups after operation (n = 20).

Isokinetic motion mode	Group-A	Group-B	Group-C	p Value
Abduction (N*m/kg)	0.59 ± 0.07	0.55 ± 0.11	0.41 ± 0.09	0.001
Adduction (N*m/kg)	0.71 ± 0.14	0.67 ± 0.08	0.52 ± 0.07	0.012
Flexion (N*m/kg)	0.75 ± 0.05	0.73 ± 0.12	0.56 ± 0.10	0.021
Extension (N*m/kg)	1.21 ± 0.19	1.16 ± 0.22	0.97 ± 0.13	0.042

## DISCUSSION

A comparative analysis of PT/BW during abduction, adduction and flexion of the hip joint on the affected side amongst different groups showed that it was the lowest in Group-C (severe coxa varus deformity) and lower than that in Group-A (femoral varus deformity) and Group-B (mild coxa varus deformity). These findings were similar to those reported by Horstmann;^6^ that is, severe hip osteoarthritis might exhibit a remarkable effect on the function of the muscle Group-around the hip joint. All these data suggest that in patients with FD of the proximal femur, serious varus deformity before operation may be associated with evident muscle dysfunction around the hip joint after operation. As reported by Casartelli et al.^7^, the degree of deformity was the main risk factor affecting the recovery of postoperative function, and gait function would frequently have poor recovery when the neck-shaft angle was <60° in patients with coxa varus deformity. However, preoperative muscle strength comparison is lacking, which would be impossible to indicate the improvement of postoperative muscle strength before operation. Moreover, muscle strength compared with the healthy side revealed unsatisfactory recovery on the affected side, indicating the presence of varying degrees of muscle dysfunction around the hip in the studied patients.

FD, also known as FD of bones, was named for the first time by Lichtenstein and Jaffe in 1938 and 1942. It is a non-hereditary tumour-like lesion that is primarily characterised by the replacement of healthy bone tissue with dysplastic fibrous tissue.^8^ Proximal femur is the predilection site of FD.^9^ Zhang et al.^10^ reported that 92% of the cases involved the femurs in patients with multiple FD. Meanwhile, the proximal femur is the site where the stress is most concentrated. In case the normal bone trabecular structure is replaced by abnormal fibrous tissue, the local bone anatomical structure may be destructed, and the mechanical strength may be reduced, leading to the potential risk of miniature pathological fracture. Furthermore, under the action of its own stress, the femoral neck bends and the neck-shaft angle decreases to induce the formation of coxa varus deformity.

In addition, when the lesion is located below the trochanter, the femoral shaft tends to bend because of the traction of the hip muscle, causing displacement of the force line laterally to induce femoral varus deformity. Corresponding clinical manifestations are limb reduction defects, claudication, joint pain, pathological fracture, etc., which seriously affect the daily life of patients.^11^ At present, existing therapeutic options clinically include osteotomy, curettage, bone grafting and internal fixation, aiming to correct the deformity and restore the length and force line of the affected limb. However, less attention has been paid to the reconstruction and recovery of muscle and soft tissue around the hip.

Isokinetic muscle strength testing: isokinetic muscle strength training and testing system is a novel approach for muscle function evaluation and muscle strength training. Based on its basic principle, this test can be performed by using a proper instrument to generate resistance matched with muscle power in real time, achieve the balance of torque and maintain constant speed.^12^ PT is the index for evaluating muscle strength in a single movement direction in the isokinetic muscle strength training and testing system, which has also been recognized as the ‘gold standard’.^13^,^14^ For example, Rossi et al.^15^ carried out isokinetic muscle strength testing and joint function scoring on 20 patients after single hip arthroplasty. The result showed that the proposed system can accurately reflect the function after hip arthroplasty, which can be considered a new index to evaluate the soft tissue function after the treatment of hip osteoarthritis.

The decrease of muscle strength around the hip after operation has four main causes. Firstly, degeneration and dysfunction of muscle and soft tissue around the hip and joint instability occurred before operation. Meanwhile, in a study by Ippolito et al.^16^, 27 cases (five years old at the initial diagnosis) of femoral FD with deformity treated conservatively were followed up for seven years. After three years of follow-up, 61% of the children showed deformity progression, and 87% of the children showed significantly aggravated deformity, thereby leading to reduced muscle strength of the affected limb and poor postoperative recovery at seven years of follow-up. Secondly, patients may have intraoperative muscle and soft tissue injuries. In patients with FD of the proximal femur with varus deformity, the osteotomy is mostly performed in the region of the trochanter and proximal femoral shaft. In accordance with our hypothesis, the results confirmed the lowest index of muscle strength around the hip in Group-C. Maurer et al.^17^ also reported that trochanteric osteotomy led to poor recovery of muscle strength in patients with ‘shepherd’s crook’ deformity aged less than 18 years.

Thirdly, the initial length of the muscle changed. The neck-shaft angle may decrease in patients with coxa varus, which may induce a variation of the spatial position of the greater trochanter and spatial displacement in the insertion of the attached muscle, thereby affecting the initial length of the muscle and muscle strength correspondingly. Fourthly, a timely and effective muscle function exercise was not carried out in these patients postoperatively. Inactivity and improper activity for a long time may result in muscle dysfunction.

### Limitations of this study:

The number of subjects included in this study is limited, and we only analyzed the cases operated in our hospital, which may not be representative enough. We look forward to a multi-center study in the future to reach more comprehensive conclusions.

## CONCLUSIONS

The muscle function around the hip joint plays an important role in the improvement of the proximal femur and hip joint function and restoration of gait after orthopaedic osteotomy.^18^ Moreover, apart from the bone and limb force line, improving the muscle function around the hip is necessary, which should be regarded as an important part of the treatment of FD of the proximal femur with varus deformity.

### Authors’ Contributions:

**ZL:** Designed this study and prepared this manuscript, are responsible and accountable for the accuracy and integrity of the work.

**XZ:** Collected and analyzed clinical data.
